# Clinical and Genetic Aspects of CADASIL

**DOI:** 10.3389/fnagi.2020.00091

**Published:** 2020-05-07

**Authors:** Toshiki Mizuno, Ikuko Mizuta, Akiko Watanabe-Hosomi, Mao Mukai, Takashi Koizumi

**Affiliations:** Department of Neurology, Graduate School of Medical Science, Kyoto Prefectural University of Medicine, Kyoto, Japan

**Keywords:** CADASIL, notch signaling, GOM, white matter lesion, vascular dementia

## Abstract

Cerebral autosomal dominant arteriopathy with subcortical infarcts and leukoencephalopathy (CADASIL), a hereditary cerebral small vessel disease caused by mutations in *NOTCH3*, is characterized by recurrent stroke without vascular risk factors, mood disturbances, and dementia. MRI imaging shows cerebral white matter (WM) hyperintensity, particularly in the external capsule and temporal pole. Missense mutations related to a cysteine residue in the 34 EGFr on the NOTCH3 extracellular domain (N3ECD) are a typical mutation of CADASIL. On the other hand, atypical mutations including cysteine sparing mutation, null mutation, homozygous mutation, and other associate genes are also reported. From the viewpoint of gain of function apart from Notch signaling or loss of function of Notch signaling, we review the research article about CADASIL and summarized the pathogenesis of small vessel, stroke, and dementia in this disease.

## Introduction

**Figure 1:**
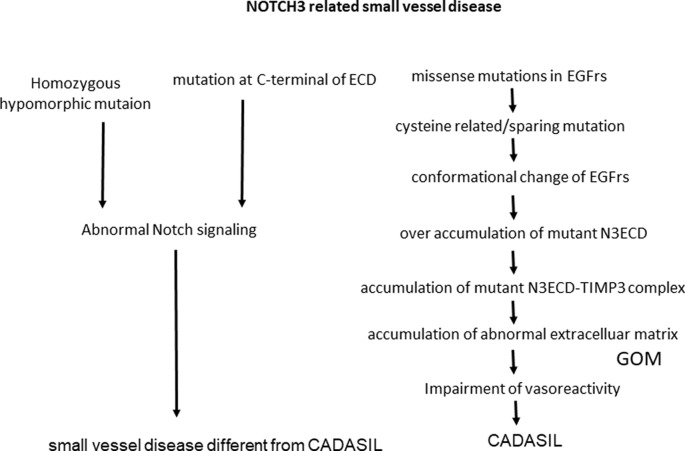
NOTCH3 is related to small vessel disease. ECD, extracellular domain; N3ECD, Notch3 extracellular domain; EGFrs, Epidermal Growth Factor repeats; GOM, granular osmiophilic material; TIMP3, tissue inhibitor of metalloproteinases 3.

Cerebral autosomal dominant arteriopathy with subcortical infarcts and leukoencephalopathy (CADASIL) is one of the most common hereditary cerebral small vessel diseases caused by mutations in *NOTCH3*. C was first recognized as a clinical and genetic hereditary small-vessel disease entity that induces cerebral infarction, white matter (WM) disease, microbleeds, and finally, vascular dementia. Understanding the impact of this disease is important for analyzing small-vessel diseases as well as vascular dementia. This review article is summarized in terms of whether CADASIL is caused by the gain of NOTCH3 function or by loss of NOTCH signaling function. Supporting data for a gain of function is pro-aggregatory property of cysteine related or cysteine-sparing *NOTCH3* mutations as well as homozygous *NOTCH3* mutation. Also, pathological findings of the accumulation of extracellular matrix and granular osmiophilic material (GOM) supported the hypothesis that Notch extracellular domain (NECD) can be a core of aggregation. Looking from the other side, nonsense *NOTCH3* mutation or constitutive activation of NOTCH3 signaling can be related to the pathogenesis of different cerebral small vessel disease from CADASIL (Figure 1).

## Genetics

### *NOTCH3* Mutations

Notch signaling is evolutionarily conserved, and four members of the Notch receptor family: NOTCH1-4 exist in humans. *NOTCH3* encodes a single-pass transmembrane receptor, NOTCH3, which consists of a NECD including 34 epidermal growth factor-like repeats (EGFrs) and an Notch intracellular domain (NICD). The Notch protein is thought to undergo complex proteolytic processing events. The first cleavage, named as S1 cleavage of the receptor occurs on the Golgi for the maturation of the receptor (i.e., the formation of the NECD-NICD heterodimer). The second proteolysis, named as S2 cleavage by A disintegrin and metalloprotease 10 (ADAM10), occurs on the cell surface when the receptor interacts with NOTCH ligand, Jagged or Delta/Serrate/LAG-2 (DSL), on neighboring cells (Brou et al., 2000). The third cleavage, named as S3 cleavage: γ-secretase–dependent intra-membrane proteolysis, NICD is moved into the cytoplasm and shuttles to the nucleus. Finally, NICD activated target genes with transcriptional cofactors of the CBF1-Su(H)-Lag1 (CSL) family (Bray, 2006). While *NOTCH3* mainly expressed in the central nervous system in the fetus, *NOTCH3* is expressed predominantly in vascular smooth muscle cells (VSMCs) to maintain vascular contractility in adults (Joutel et al., 2000).

## *NOTCH3* Mutations in Cadasil Patients

### Cysteine-Related

To date, more than 200 cysteine-related mutations, most of which are single nucleotide changes, have been reported (Supplementary Table S1, Joutel et al., 1997; Rutten et al., 2014; Koizumi et al., 2019; Leiden Open Variation Database, or references therein). A few exceptions include in-frame insertion/deletion mutations and splicing-site mutations (Tikka et al., 2009). Also, a rare mutation of in-frame 15 bp duplication in exon 7 is reported (Lee et al., 2011). CADASIL-associated mutations are localized from exon2 to 24, which encode EGFrs. Each EGFr contains six cysteine residues that likely participate in forming three pairs of disulfide bonds to maintain the normal NOTCH3 protein conformation. Most of the mutations are of the missense type, resulting in an even number to an odd number of cysteine residues (Joutel et al., 1997; Mizuta et al., 2017). The resulting unpaired cysteine is predicted to cause abnormal disulfide bridge formation that leads to aggregation of NECD (Duering et al., 2011).

The mutations accumulate in EGFrs 1–6, apart from the ligand-binding domain, EGFrs 10 and 11. Cellular experiments showed that most of the mutations do not affect Notch signaling, suggesting that CADASIL is not caused by signaling dysfunction. However, NOTCH3 harboring a p.Cys428Ser mutation in EGFr 10 and p.Cys455Arg in EGFr 11 exhibits attenuated ligand-binding activity, resulting in a significant reduction of NOTCH3 signaling (Joutel et al., 2004; Peters et al., 2004b). A previous report showed that mutations in EGFrs 10 and 11 are associated with milder cognitive deficits and a trend toward a lower volume of lacunar infarcts compared with the common mutations in EGFrs 2–5 (Monet-Leprêtre et al., 2009). This association remains to be elucidated, but it is possible that Notch signaling may affect the clinical symptoms.

Cases with duplication (Lee et al., 2011) or deletion of *NOTCH3* (Dichgans et al., 2000) are reported. These mutations also change the number of Cysteine residue as well as point mutation and GOM was detected in the cases with these mutations (Lee et al., 2011).

### Biological Effect of CADASIL-Associated Mutations

A popular hypothesis holds that the *NOTCH3* mutations causing CADASIL are gain-of-function rather than loss-of-function mutations (Carare et al., 2013). One of the evidence supporting this hypothesis was the identification of hypomorphic mutations in individuals without the CADASIL phenotype. Rutten et al. (2013) reported two *NOTCH3* nonsense mutations, c.307C>T, p.Arg103*, in two brothers aged in their 50s; brain MRI and skin biopsy results showed incompatible with CADASIL. Also, they reported a CADASIL patient with compound heterozygous for a pathogenic *NOTCH3* mutation, p.Tyr710Cys, and an intragenic frameshift deletion. In that patient’s family, p.Tyr710Cys segregated with the affected parent, whereas the intragenic frameshift deletion was also identified in the normal parent of the patient. They concluded that these hypomorphic NOTCH3 alleles do not cause CADASIL (Rutten et al., 2013). According to previous case reports, complete loss of, and also constitutive activation of NOTCH3 signaling are thought to cause arteriopathy. Pippucci et al. (2015) reported a 24 years-old man with childhood-onset arteriopathy and cavitating leukoencephalopathy. Exome analysis of the patient and his consanguineous parents identified homozygous NOTCH3 null mutation c.C2898A (p.C966*) in the patient. Fouillade et al. (2008) reported a 53-year-old woman with 35-years-onset stroke and MRI finding of WM hyperintensity. They identified *NOTCH3* c.4544T>C resulting in p.L1515P mutation which localizes in the C-terminal end of NOTCH3 extracellular domain (N3ECD). Although the precise mechanism remains unknown, cellular experiments suggested increased NOTCH3 signaling of this mutation in a ligand-independent manner (Pippucci et al., 2015). It is of note that the pathological hallmark of CADASIL, GOM, was not detected in either of these patients (Fouillade et al., 2008; Pippucci et al., 2015), suggesting that arteriopathy related to abnormal NOTCH3 signaling is different from CADASIL. In a study using a transgenic mouse model of CADASIL, Joutel described the toxic gain-of-function properties of mutant NOTCH3. Mice harboring human *NOTCH3* with p.Arg90Cys, p.Cys428Ser, or p.Arg169Cys mutations exhibited CADASIL-like pathologic changes, N3ECD accumulation, and deposition of GOM (see below). Notably, transgenic mice with p.Cys428Ser, which cannot mediate Notch signaling (see above), also showed GOM deposition, suggestive of toxic gain-of-function mutations leading to aggregation of mutated N3ECD (Joutel, 2011).

### Cysteine-Sparing Mutations

Muino et al. (2017) recently reviewed cysteine-sparing *NOTCH3* missense mutations and proposed criteria for their pathogenicity: (a) typical clinical CADASIL syndrome; (b) diffuse white matter hyperintensity (WMH); (c) 33 NOTCH3 exons analyzed; (d) mutations that were not polymorphisms; and (e) GOM deposits noted in skin biopsy. Of the 25 mutations reviewed, they concluded that p.Arg61Trp, p.Arg75Pro, p.Asp80Gly, and p.Arg213Lys fulfilled these criteria. To uncover the pathogenicity of cysteine-sparing mutations, analysis of pro-aggregatory property may be important. Some cysteine-involving mutations (Duering et al., 2011) and also p.Arg75Pro and p.Asp80Gly (Wollenweber et al., 2015) was reported to be prone to aggregation by using a single-particle aggregation assay. In the very recent report of the patient with p.Gly73Ala, unfortunately, skin biopsy was declined, cellular and *in vitro* experiments showed pro-aggregatory property of the mutation (Huang et al., 2020).

Of the pathogenic cysteine-sparing mutations, p.Arg75Pro is probably the most frequent, and this mutation is primarily reported in eastern Asians (Kim et al., 2006; Mizuno et al., 2008; Ueda et al., 2015). In addition to positive skin biopsy findings, we also noted co-segregation in a p.Arg75Pro family and demonstrated the predicted conformational change in the EGFr harboring this mutation (Mizuno et al., 2008). Japanese CADASIL patients with p.Arg75Cys exhibit atypical and mild phenotypes, including a lower frequency of stroke/TIA and temporal pole lesions, with a tendency toward an older age at onset (Ueda et al., 2015; Koizumi et al., 2019).

### Homozygous Mutations

Although CADASIL is generally caused by heterozygous mutations in *NOTCH3*, several CADASIL cases involving homozygous mutations have been reported (Mukai et al., 2018). Interestingly, some cases involving homozygous mutations showed a more-severe clinical phenotype than cases involving heterozygous mutations, but other cases were within the spectrum of the heterozygous phenotype.

The most frequent homozygous mutation is p.Arg544Cys. Mukai et al. (2018) reported a 63-year-old male case presenting first stroke attack with only mild weakness of the left leg and recovered well, involving a homozygous p.Arg544Cys mutation; GOM around the basement membrane of VSMCs on skin biopsy were detected in this case. No other stroke patients were detected in his parents and siblings indicated the tendency toward a mild phenotype with the p.Arg544Cys mutation in agreement with previous reports (Liao et al., 2015; Lee et al., 2016). p.Arg544Cys locates not in EGFr, but between the 13th and 14th EGFrs. Therefore, we hypothesize that p.Arg544Cys may contribute to a milder effect to a conformational change of EGFr resulting in a milder phenotype. The p.Arg544Cys mutation is also notable because of its geographic accumulation. The frequency of this mutation in CADASIL patients is 93.6% in Jeju Island, Korea, and 70.5% in Taiwan (Liao et al., 2015; Lee et al., 2016).

## Disease-Modifying Genes

### Genome-Wide Association Study of CADASIL

Opherk et al. (2014) performed a genome-wide association study to identify genetic modifiers of WMH volume in CADASIL. They analyzed SNP array data for 466 patients and found no SNPs reaching genome-wide significance. However, polygenic score analyses which included SNPs with weak *p*-values, indicated significant association with WMH volume when 10,574 SNPs (each *p*-value < 0.1) or 52,125 SNPs (each *p*-value < 0.5) were included. They suggested that multiple variants exert small effects on the WMH burden in CADASIL.

### RNF213

We recently reported ring finger protein 213 (RNF213)-related susceptibility to intracranial arterial stenosis (ICAS) in CADASIL patients (Yeung et al., 2018). The frequency of RNF213 variants was 23.5% in CADASIL patients with ICAS, compared with 1.9% in those without ICAS. CADASIL is recognized as a small-vessel disease, but intracranial major artery stenosis was reported in some CADASIL patients (Choi et al., 2005). The susceptibility variant rs112735431, c.14576G>A (p.R4859K) or c.14429G>A (p.R4110K) in RNF213, was originally identified as prominently associated with moyamoya disease, mainly reported in eastern Asia. Miyawaki et al. (2012) found the variant was linked to susceptibility to ICAS even in sporadic cases. These associated genes may contribute to the clinical phenotypes of CADASIL.

## Pathomechanism From the Perspective of Mutation and Pathology

### Pathology

CADASIL affects small vessels in the brain WM and deep gray matter, resulting in thickening of vascular walls and luminal stenosis. In the tunica media, degeneration of VSMCs, positive PAS staining, and granular deposits of N3ECD immunoreactivity are observed (Baudrimont et al., 1993; Joutel et al., 2000). In the tunica adventitia, accumulation of various fibrous extracellular matrices is observed, including collagen, laminin, and clusterin. However, vessel occlusion or thrombosis is rarely found (Ruchoux et al., 1995). Therefore, the direct pathogenic mechanism leading to lacunar infarction in CADASIL remains to be elucidated. A popular hypothetical pathomechanism involves hemodynamic disturbance of lesion-affected arterioles and loss of compliance and autoregulation (Tikka et al., 2014).

### GOM

The first ultrastructural descriptions of perivascular deposits surrounding small, penetrating arteries in the brain, designated GOM, were reported by Baudrimont et al. (1993). This material has been examined mainly in the brain, but GOM is also found surrounding VSMCs in other tissues, including muscle and skin (Ruchoux et al., 1995). Tikka et al. (2009) investigated the GOM in CADASIL patients and concluded that COM is specific to CADASIL. Immunohistochemistry and immunogold electron microscopy studies revealed the distribution of GOM and N3ECD protein in the microvasculature of brain gray matter and WM. Immunogold electron microscopy using an antibody to N3ECD revealed abundant particles in the GOM within microvessels, VSMC membranes, and perivascular cells (Yamamoto et al., 2013). These results suggest that NOTCH3 fragments are major components of GOM deposits.

### Transendocytosis of NOTCH3

Cisendocytosis of either the DSL ligands or the Notch receptor itself into the cytoplasm has been recognized as playing an important role in regulating Notch signaling (Bray, 2006; Fortini and Bilder, 2009; Pratt et al., 2011). Also, genetic and cellular biological studies have shown that Notch is endocytosed into neighboring ligand-expressing cells in *Drosophila* (Klueg and Muskavitch, 1999) and that endocytosis of NECD promotes Notch proteolysis and downstream signaling in mammals (Nichols et al., 2007). Therefore, endocytosis of NECD into ligand-expressing cells, known as trans-endocytosis, is believed to be more critical for Notch activation than proteolytic events (Nichols et al., 2007). Because CADASIL causing mutations localize in N3ECD, we hypothesized that impairment of N3ECD trans-endocytosis may be a pathological mechanism of CADASIL. We addressed this issue by using HEK293 cells harboring a single copy of mutant or wildtype human *NOTCH3* cDNA cocultured with Jagged1-expressing cells (Watanabe-Hosomi et al., 2012). In this co-culture system, Notch signaling quantified by HES1 expression was similar between mutant and wildtype N3ECD, in agreement with previous reports. However, we found that C185R mutant N3ECD on the cell surface is degraded significantly more slowly than wild-type N3ECD in NOTCH3 cells. While vesicles containing N3ECD were observed in Jag1-expressing cells co-cultured with wild-type NOTCH3, vesicles with mutant N3ECD within the Jag1-expressing cells were significantly fewer in number. These results indicate that the process of degradation of mutant N3ECD on the cell surface is disturbed due to the impairment of trans-endocytosis (Watanabe-Hosomi et al., 2012). It can also explain the abnormal accumulation of N3ECD in vascular walls without accumulation of the intracellular domain of Notch3 intracellular domain (N3ICD) or full-length protein, and signaling is activated normally. Further approach is necessary to uncover the process between impaired trans-endocytosis and abnormal accumulation of N3ECD and GOM deposit.

### TIMPS3 and VTN

Using cultured cells, Monet-Leprêtre et al. (2013) provided evidence that excess levels of or multimerization of mutant Notch3 ECD facilitate interactions with key components of the vascular extracellular matrix, including tissue inhibitor of metalloproteinases 3 (TIMP3) and VTN. Brain vessels from transgenic mice and patients with CADASIL exhibit elevated levels of both insoluble cross-linked and soluble TIMP3 species (Monet-Leprêtre et al., 2013). Later, Capone et al. (2016) showed that reducing TIMP3 or VTN ameliorated CADASIL phenotype using transgenic mice, suggesting TIMP3 or VTN may be a novel therapeutic target of CADASIL.

### Downstream Signaling Related to TGF-β

It is well known that transforming growth factor-β (TGF-β) signaling is important in the regulation of fibrotic events in vessels and other various tissues. Increased TGF-β signaling was reported as the pathophysiology of cerebral autosomal recessive arteriopathy with subcortical infarcts and leukoencephalopathy (CARASIL; Hara et al., 2009), suggesting TGF-β signaling as a key pathway to cerebral small vessel diseases. Kast et al. (2014) examined molecules involving in the regulation of TGF-β bioavailability, fibronectin, fibrillin-1, and latent TGF-β–binding protein 1 (LTBP-1), in post-mortem brain tissue from CADASIL patients. All the three molecules were enriched in the CADASIL vessel. However, fibronectin and fibrillin-1 did not colocalize with N3ECD deposits, whereas, LTBP-1 showed a striking co-localization with N3ECD deposits, suggesting specific recruitment of LTBP-1 into aggregates. Also, increased levels of the TGF-β prodomain indicate dysregulation of the TGF-β pathway in CADASIL development. *In vitro* co-aggregation assay showed a direct interaction between LTBP-1 and mutant N3-ECD but no interaction between LTBP-1 and wildtype N3-ECD. These suggested a specific co-aggregation of LTBP-1 with mutant NOTCH3 and possible TGF-β signaling impairment in CADASIL (Kast et al., 2014).

### Reconstruction of Small Vessels

To examine the degree and extent of the pathologic changes, Okeda et al. (2002) analyzed the entire length of vessels by reconstructing 1,000 serial sections of the 11 cerebral medullary arteries in an autopsy of a CADASIL patient who was 75 years old. The predominant findings were loss of VSMCs in the tunica media and fibrosis in the tunica adventitia. Most arteries exhibited continuous complete loss of VSMCs in the WM. Severe adventitial fibrosis was found in all arteries but restricted to WM. However, no stenosis or occlusion was found in the arteries studied. Considering their results collectively, they used a “so-called earthen pipe state” to describe the state of lesioned arteries and proposed failure of autoregulation of cerebral blood flow due to the earthen pipe state as underlying the pathogenesis of CADASIL (Okeda et al., 2002).

### Pericytes

As the receptor protein encoded by the *NOTCH3* gene is expressed not only on VSMCs but also on pericytes, pericytes and capillary vessels can be damaged by CADASIL (Dziewulska and Lewandowska, 2012). Degeneration and loss of pericytes in capillary vessels were detected in the microvessels in the autopsy of the brain and skin-muscle biopsy in CADASIL patients (Dziewulska and Lewandowska, 2012). GOM was usually seen near pericyte cell membranes or within infoldings (Dziewulska and Lewandowska, 2012). These findings suggested increased permeability of the capillary vessels and disturbances in cerebral microcirculation; this degeneration can also cause defective vasomotor reactivity in CADASIL (Okeda et al., 2002; Qin et al., 2019).

### Why Clinical Symptoms Occur Only in the Brain

Because NOTCH3 is expressed ubiquitously in VSMCs, it is not surprising that pathological changes including GOM can be detected not only in the brain but also in other organs. It is reported that peripheral vascular function, as well as in the brain, was also impaired in patients with CADASIL and also a transgenic mice model of CADASIL (Fujiwara et al., 2012).

Clear phenotypes are restricted to the brain, but several studies suggested cardiovascular (van den Boom et al., 2003; Rufa et al., 2007) and renal (Kusaba et al., 2007; Guerrot et al., 2008; Ragno et al., 2012) involvement, though their causal relationship is still unclear. A recent article by Kelleher et al. (2019) showed that CADASIL iPSC-derived mural cells, including VSMCs and pericytes were susceptible to apoptotic stress. This may explain why major symptoms are restricted to the brain, because the blood-brain barrier, the crucial structure to maintain brain homeostasis, consists of astrocytes and pericytes (Ihara and Yamamoto, 2016).

## Clinical Features of Cadasil

### Incidence

To date, thousands of families with CADASIL have been diagnosed worldwide in many different ethnic groups. The disorder is often overlooked and misdiagnosed. Its minimum prevalence has been estimated at between 2 and 5 in 100,000 but may vary between populations (Razvi et al., 2005; Narayan et al., 2012; Moreton et al., 2014; Bianchi et al., 2015).

### Genotype-Phenotype Correlation

There have been a limited number of studies involving genotype-phenotype analyses, some of which reported negative results (Adib-Samii et al., 2010). On the other hand, a German study including 371 CADASIL patients showed positive findings in genotype-phenotype analyses of eight of the most frequent genotypes: p.Arg90Cys, p.Cys117Phe, p.Arg133Cys, p.Arg14.

1Cys, p.Arg153Cys, p.Arg169Cys, p.Cys174Tyr, and p.Arg182Cys (Opherk et al., 2004). Both p.Cys174Tyr and p.Cys117Phe was significantly associated with a lower median age at death, and p.Cys117Phe alone was significantly associated with a lower median age at onset of stroke and immobilization (Opherk et al., 2004). Genotype-phenotype correlation analyses of each mutation should be carefully conducted due to the wide phenotype distribution, even in the same family harboring the same mutation.

### EGF Repeats 1–6 vs. 7–34

Rutten et al. (2019) recently demonstrated the effect of mutation location on the severity of the disease. By comparing CADASIL patients with mutations in EGFr 1–6 and EGFr 7–34, those in the EGFr 1–6 group had a 12-year earlier onset of stroke, lower survival, and higher WMH volume than those in the EGFr 7–34 group. As described above, *NOTCH3* mutations in CADASIL patients accumulate in EGFr 1–6, at 71.8% in Europeans (Rutten et al., 2019). It is noteworthy that *NOTCH3* pathogenic variants were identified in a recent analysis of a large-scale genome variation database of a general population (Rutten et al., 2016). *NOTCH3* pathogenic variants in a general population accumulated in EGFr 7–34, at 97.5% in the general European population, based on the Genome Aggregation Database (Rutten et al., 2016). Taken together, the results of Rutten et al. (2019) suggest a predisposition toward EGFr 1–6 in the classical, more-severe CADASIL phenotype. They also suggest a broad disease spectrum involving EGFr 7–34, from a mild phenotype to possible non-penetrance, and these results highlight the significant role of *NOTCH3* pathogenic variants in general populations.

### Environmental Factors

The difference between clinical symptoms and course in the same family harboring the same *NOTCH3* mutation indicates the importance of environmental factors. Of the two twin studies of CADASIL, the first reported one found apparent different phenotypes, including 14 years difference of the age at onset between the twins, suggesting an effect of environmental factors on the disease (Mykkänen et al., 2009). The second reported one found similar phenotypes, including the age at onset of 74–75 years old and parkinsonism in the twin (Ragno et al., 2016). A similar phenotype indicates similar lifestyle-related factors, and Ragno’s cases did not contradict the possible effect of environmental factors on the CADASIL phenotype (Ragno et al., 2016). Conventional vascular risk factors may influence the severity of the disease. Adib-Samii et al. (2010) analyzed 200 symptomatic CADASIL patients in the UK; they found that hypertension (odds ratio 2.57) and pack-years of smoking (odds ratio 1.07) were associated with an increased risk of stroke. *Ciolli* also assessed the influence of vascular risk factors and revealed that hypertension was related to both disability assessment for dementia (DAD) score and disability (Ciolli et al., 2014). In our cohort, 62.9% of CADASIL patients had vascular risk factors, including hypertension, diabetes mellitus, hyperlipidemia, smoking, or alcohol consumption, and they were more prone to stroke (Mizuta et al., 2017). Management of these factors are essential in CADASIL as well as sporadic cases.

## Diagnosis

### Criteria for CADASIL Diagnosis

Original criteria for CADASIL diagnosis were proposed by Davous in 1997 (Davous, 1998). This precious work contributed to the core concept of CADASIL when CADASIL was not well recognized by physicians yet. However, genetic tests of *NOTCH3* revealed atypical cases, and the clinical phenotype of CADASIL was diverse, ranging from asymptomatic to severe. The *Davous’s* criteria cannot be applied to atypical CADASIL cases because the criteria are strict and have low sensitivity. In particular, cases involving elderly onset, no family history, or positive cardiovascular risk factors might be overlooked using these criteria. To avoid missing suspected CADASIL patients before genetic testing, we proposed more-sensitive criteria from Japanese CADASIL cases (Mizuta et al., 2017; Supplementary Table S2). The sensitivity of our new criteria is 97%, sufficient to screen candidates for CADASIL and to aid genetic testing (Mizuta et al., 2017).

### CADASIL Scale and CADASIL Scale-J

To prioritize access to genetic testing for suspected CADASIL patients, a quantitative evaluation of CADASIL-specific features possessed by each patient is necessary. Markus proposed a diagnostic procedure based on skin biopsy and involvement of the anterior temporal lobe on MRI (Markus et al., 2002). As pre-genetic screening approaches are desirable, Pescini et al. (2012) developed the CADASIL scale, a screening tool applied in the clinical setting due to the high cost and time-consuming nature of genetic testing. The weighted scores to common disease features based on frequencies obtained in a pooled analysis of selected international CADASIL series. The cut-off score of the definitive CADASIL scale had a sensitivity of 96.7% and specificity of 74.2%. Unfortunately, the sensitivity of the CADASIL scale was 52.1% in our Japanese cohort because of some clinical differences about the CADASIL group as well as NOTCH3-negative CADASIL-like patients (non-NOTCH3) group between the European population and the Japanese population (Koizumi et al., 2019). Several studies revealed a low prevalence rate of migraine in Japanese CADASIL compared to European CADASIL patients (Uchino et al., 2002). Although WM lesion at the temporal pole is specific CADASIL patients in both cohort, WM lesion at the external capsule was different in the non-NOTCH3 group in each cohort. These differences decreased the sensitivity and specificity of the original CADASIL scale when applied to the Japanese cohort. Therefore, we modified the CADASIL scale based on the clinical features of 126 Japanese CADASIL patients and 53 Non-NOTCH3 to develop CADASIL scale-J (Supplementary Table S3; Koizumi et al., 2019). In CADASIL scale-J, the score ranged from 0 to 25 and a cut-off value of 16, using eight items. The sensitivity and specificity of the CADASIL scale-J were enough quality for prioritizing tool before genetic testing.

## Imaging

### White Matter Lesions

MRI can visualize the characterization of WM lesions and lacunar infarction in stroke syndromes. In CADASIL, T2-weighted hyperintensity was noted in the deep WM, internal and external capsules, and the temporal pole. Temporal pole hyperintensity on T2-weighted and FLAIR MR sequences can be detected even in the early 20s of CADASIL patients (Chabriat et al., 1998; Mizuno, 2012). Dilated perivascular spaces (PVS) in CADASIL patients are located in the lentiform nuclei (94%) and subcortical WM of the temporal lobes (66%; Chabriat et al., 1998). PVS around small perforating arteries are pial-lined, interstitial fluid-filled spaces, readily seen to be enlarged in the WM of elderly subjects. Yamamoto et al. (2009) performed a postmortem study to quantify the degree and extent of PVS and arteriopathic changes within the temporal pole WM of CADASIL subjects. They concluded that the MRI hyperintensity in the temporal pole of CADASIL patients could be explained by enlarged PVS and degeneration of myelin rather than lacunar infarcts.

### Lacunar Infarction

Viswanathan analyzed clinical data from 147 consecutive patients and revealed a significant independent association between age, volume of lacunar lesions, and global cognitive function scales, although WMH and microbleeds had no independent influence on cognitive function. Disability was associated with the volume of lacunar lesions, microbleeds, systolic blood pressure, and age but not with WMH (Viswanathan et al., 2007). Liem et al. (2007) analyzed 62 symptomatic and 15 asymptomatic members of CADASIL and revealed that the severity of cognitive dysfunction in mutation carriers is independently associated with MRI infarct lesion load. In contrast, WMH lesion load and microbleeds were not associated with cognitive dysfunction after correcting for age. These data indicate the importance of lacunar infarction in the progression of CADASIL.

Viswanathan examined the relative impact of lesion burden and location of these MRI markers on cognitive impairment and disability combined with whole-brain mean apparent diffusion coefficient (mean-ADC) and brain parenchymal fraction (BPF). In multivariate models accounting for lesion burden and location, the volume of lacunar lesion, mean-ADC, and BPF each had an independent influence on global cognitive function and disability (Viswanathan et al., 2010). Particularly, brain atrophy was shown to have the strongest independent influence on clinical impairment in CADASIL when all MRI markers in the disease are considered together. These results suggest that the clinical impact of cerebral cortical loss is important for CADASIL dementia (Viswanathan et al., 2010).

### 7-T MRI Reveals Micro-cortical Infarction

Jouvent et al. (2011b) used high-resolution postmortem 7-T MRI to examine infarcts of the cerebral cortex in a CADASIL patient with pathology examination. These lesions were not visible on the *in vivo* MRI obtained at 1.5 T. They examined cortex morphology and clinical worsening in 190 CADASIL patients and showed that reduction of sulcal depth is independently associated with increased time to complete trail making test A and B and that of cortical thickness to increased disability. They also showed that the impact of volume of lacunar lesions on cortical changes is greater than that of the volume of WMH and that cortical changes related to lacunar lesions evolve parallel to clinical worsening. These results support the hypothesis that cortical changes in CADASIL play a role in disease pathophysiology (Jouvent et al., 2011a).

### Natural History

Few prospective studies have examined the natural history of CADASIL. Davous summarized the natural history of 134 documented cases and showed a mean age at onset of 40.3 years. The mean duration of the disease was 13.6 years, and the mean age at death was 56.7 years (Davous, 1999). *Opherk* summarized the natural history of a larger CADASIL cohort and reported a median age at onset of stroke of 50.7 years in men and 52.5 years in women, the median age at death of 64.6 years in men and 70.7 years in women (Opherk et al., 2004). In our cohort including 200 CADASIL patients, the mean age at onset of stroke was 48.3 years in men and 52.2 years in women (Koizumi et al., 2019). These results indicated a similar clinical course in any ethics, but more studies need to clarify a difference in each ethics before starting a disease-modifying-therapy on CADASIL.

Peters et al. (2004a) reported that their cohort deteriorated for all clinical scales over 2 years. There were 18 strokes within 173 person-years, giving an average incidence rate of stroke of 10.4 per 100 person-years. Age at baseline was found to be a predictor of clinical progression (Peters et al., 2004a). These data indicate a younger age of onset of stroke and death compared with the general population. CADASIL researchers should thus develop new approaches to improve this natural history.

## Author Contributions

TM and IM conceived the original idea. TM wrote the manuscript with support from IM, AW-H, MM, and TK.

## Conflict of Interest

The authors declare that the research was conducted in the absence of any commercial or financial relationships that could be construed as a potential conflict of interest.

## Abbreviations

ADAM10: A disintegrin and metalloprotease 10
ADC: apparent diffusion coefficient
BPF: brain parenchymal fraction
CADASIL: cerebral autosomal dominant arteriopathy with subcortical infarcts and leukoencephalopathy
CARASIL: cerebral autosomal recessive arteriopathy with subcortical infarcts and leukoencephalopathy
CM: cerebral microbleeds
CSL: CBF1-Su(H)-Lag1
DAD: Disability Assessment for Dementia
DSL: Delta/Serrate/LAG-2
ExAC: exome aggregation consortium
EGFrs: epidermal growth factor-like repeats
ICAS: intracranial arterial stenosis
Jag1: Jagged1
LTBP-1: latent TGF-β–binding protein 1
mean-ADC: mean apparent diffusion coefficient
NECD: Notch extracellular domain
NICD: Notch intracellular domain
N3ECD: Notch3 extracellular domain
N3ICD: Notch3 intracellular domain
Non-NOTCH3: NOTCH3-negative CADASIL–like patients
GOM: granular osmiophilic material
PVS: perivascular spaces
RNF213: ring finger protein 213
TGF-β: transforming growth factor-β
TIMP3: tissue inhibitor of metalloproteinases 3
VSMC: vascular smooth muscle cell
VTN: vitronectin
WM: white matter
WMH: white matter hyperintensity
WML: white matter lesion.
