# Supplementary material for: Clinical and Genetic Aspects of CADASIL
Source: Front Aging Neurosci. 2020 May 7;12:91. doi: 10.3389/fnagi.2020.00091 (PMC7224236; doi:10.3389/fnagi.2020.00091)
Supplement: Supplementary file 1 [file Table_1.DOCX]

**Table 1. *NOTCH3* mutations causing CADASIL.** This list was modified from lists in Rutten et al., 2014 combined with Tikka et al., 2009 and our latest report, Koizumi et al., 2019. All the mutations are cysteine-related, except for p.Arg75Pro. Please see references therein for each mutation.

| **No in Rutten, 2014** | **No in Tikka, 2009** | ***listed in Koizumi, 2019** | **Nucleotide change** | **Amino acid change** | **Exon** | **EGFR** |
| --- | --- | --- | --- | --- | --- | --- |
| 1 | 1 |  | c.127T>G | p.Cys43Gly | 2 | 1 |
| 2 | 2 | * | c.128G>T | p.Cys43Phe | 2 | 1 |
| 3 | 3 |  | c.145T>G | p.Cys49Gly | 2 | 1 |
| 4 |  |  | c.145T>C | p.Cys49Arg | 2 | 1 |
| 5 | 4 |  | c.146G>A | p.Cys49Tyr | 2 | 1 |
| 6 | 5 |  | c.146G>T | p.Cys49Phe | 2 | 1 |
| 7 |  |  | c.157G>T | p.Gly53Cys | 2 | 1 |
| 8 | 6 | * | c.160C>T | p.Arg54Cys | 2 | 1 |
|  |  | * | c.163T>G | p.Cys55Gly | 2 | 1 |
|  |  | * | c.163T>C | p.Cys55Arg | 2 | 1 |
| 9 | 7 |  | c.179C>G | p.Ser60Cys | 2 | 1 |
| 10 |  |  | c.193T>G | p.Cys65Gly | 2 | 1 |
| 11 | 8 | * | c.194G>C | p.Cys65Ser | 2 | 1 |
| 12 | 9 | * | c.194G>A | p.Cys65Tyr | 2 | 1 |
| 13 | 10 |  | c.199T>A | p.Cys67Ser | 3 | 1 |
| 14 | 11 |  | c.200G>A | p.Cys67Tyr | 3 | 1 |
| 15 | 12 | * | c.213G>T | p.Trp71Cys | 3 | 1 |
|  | 13 | * | c.224G>C | p.Arg75Pro | 3 | 1 |
| 16 | 14 |  | c.226T>C | p.Cys76Arg | 3 | 1 |
|  |  | * | c.227G>A | p.Cys76Tyr | 3 | 1 |
| 17 | 15 |  | c.228T>G | p.Cys76Trp | 3 | 1 |
|  | 16 |  | c.231_248del | p.Gln77_Cys82del | 3 | 1 + 2 |
|  | 17 |  | c.239_253del | p.Asp80_Ser84del | 3 | 2 |
| 18 | 18 |  | c.259T>C | p.Cys87Arg | 3 | 2 |
| 19 | 19 |  | c.260G>A | p.Cys87Tyr | 3 | 2 |
|  |  | * | c.260G>T | p.Cys87Phe | 3 | 2 |
| 20 |  |  | c.265G>T | p.Gly89Cys | 3 | 2 |
| 21 | 21 | * | c.268C>T | p.Arg90Cys | 3 | 2 |
|  |  | * | c.277T>G | p.Cys93Gly |  |  |
|  | 22 |  | c.277_279dup | p.Cys93dup | 3 | 2 |
| 22 | 23 | * | c.278G>A | p.Cys93Tyr | 3 | 2 |
| 23 | 24 |  | c.278G>T | p.Cys93Phe | 3 | 2 |
| 24 |  | * | c.316T>C | p.Cys106Arg | 3 | 2 |
| 25 | 25 |  | c.318C>G | p.Cys106Trp | 3 | 2 |
| 26 | 26 |  | c.322T>C | p.Cys108Arg | 3 | 2 |
| 27 |  |  | c.323G>C | p.Cys108Ser | 3 | 2 |
| 28 | 27 |  | c.323G>A | p.Cys108Tyr | 3 | 2 |
|  |  | * | c.323G>T | p.Cys108Phe |  |  |
| 29 | 28 |  | c.324C>G | p.Cys108Trp | 3 | 2 |
| 30 | 29 | * | c.328C>T | p.Arg110Cys | 3 | 2 |
|  | 30 |  | c.341-2A>G | p.Gly114_Pro120del | intron 3 | 2 |
| 31 |  |  | c.349T>C | p.Cys117Arg | 4 | 2 |
| 32 | 31 |  | c.350G>T | p.Cys117Phe | 4 | 2 |
| 33 |  |  | c.350G>C | p.Cys117Ser | 4 | 2 |
| 34 |  |  | c.350G>A | p.Cys117Tyr | 4 | 2 |
| 35 | 32 |  | c.353C>G | p.Ser118Cys | 4 | 3 |
| 36 | 34 |  | c.368G>A | p.Cys123Tyr | 4 | 3 |
| 37 | 33 |  | c.368G>T | p.Cys123Phe | 4 | 3 |
| 38 | 36 |  | c.382T>G | p.Cys128Gly | 4 | 3 |
| 39 | 37 |  | c.383G>A | p.Cys128Tyr | 4 | 3 |
| 40 | 38 |  | c.383G>T | p.Cys128Phe | 4 | 3 |
| 41 | 39 |  | c.391G>T | p.Gly131Cys | 4 | 3 |
| 42 | 40 | * | c.397C>T | p.Arg133Cys | 4 | 3 |
| 43 | 41 |  | c.402C>G | p.Cys134Trp | 4 | 3 |
| 44 | 42 | * | c.421C>T | p.Arg141Cys | 4 | 3 |
| 45 | 43 |  | c.425T>G | p.Phe142Cys | 4 | 3 |
| 46 | 45 |  | c.431G>A | p.Cys144Tyr | 4 | 3 |
| 47 | 44 |  | c.431G>C | p.Cys144Ser | 4 | 3 |
| 48 | 46 |  | c.431G>T | p.Cys144Phe | 4 | 3 |
| 49 | 47 |  | c.434C>G | p.Ser145Cys | 4 | 3 |
| 50 | 48 |  | c.436T>C | p.Cys146Arg | 4 | 3 |
| 51 | 49 |  | c.437G>A | p.Cys146Tyr | 4 | 3 |
|  |  | * | c.438C>G | p.Cys146Trp | 4 | 3 |
| 52 | 50 |  | c.445G>T | p.Gly149Cys | 4 | 3 |
| 53 | 51 |  | c.449A>G | p.Tyr150Cys | 4 | 3 |
| 54 | 52 | * | c.457C>T | p.Arg153Cys | 4 | 3 |
|  | 53 |  | c.459_467del | p.Arg153_Cys155del | 4 | 3 |
| 55 | 54 |  | c.463T>A | p.Cys155Ser | 4 | 3 |
| 56 | 55 |  | c.464G>A | p.Cys155Tyr | 4 | 3 |
| 57 | 56 |  | c.464G>C | p.Cys155Ser | 4 | 3 |
| 58 | 56 |  | c.484T>A | p.Cys162Ser | 4 | 4 |
| 59 | 57 |  | c.484T>C | p.Cys162Arg | 4 | 4 |
| 60 | 58 |  | c.486C>G | p.Cys162Trp | 4 | 4 |
| 61 |  |  | c.493G>T | p.Gly165Cys | 4 | 4 |
| 62 | 59 | * | c.505C>T | p.Arg169Cys | 4 | 4 |
| 63 | 60 |  | c.511G>T | p.Gly171Cys | 4 | 4 |
| 64 | 61 |  | c.520T>C | p.Cys174Arg | 4 | 4 |
| 65 | 62 |  | c.520T>A | p.Cys174Ser | 4 | 4 |
| 66 | 64 |  | c.521G>A | p.Cys174Tyr | 4 | 4 |
| 67 | 63 |  | c.521G>T | p.Cys174Phe | 4 | 4 |
|  |  | * | c.521_522delinsTG | p.Cys174Leu | 4 | 4 |
| 68 | 65 | * | c.539C>G | p.Ser180Cys | 4 | 4 |
| 69 |  |  | c.542T>G | p.Phe181Cys | 4 | 4 |
| 70 | 66 | * | c.544C>T | p.Arg182Cys | 4 | 4 |
| 71 | 68 |  | c.547T>A | p.Cys183Ser | 4 | 4 |
| 72 | 67 |  | c.547T>C | p.Cys183Arg | 4 | 4 |
| 73 | 69 |  | c.548G>T | p.Cys183Phe | 4 | 4 |
| 74 | 70 |  | c.553T>C | p.Cys185Arg | 4 | 4 |
| 75 | 71 |  | c.553T>G | p.Cys185Gly | 4 | 4 |
| 76 |  |  | c.553T>A | p.Cys185Ser | 4 | 4 |
|  |  | * | c.554G>A | p.Cys185Tyr |  |  |
| 77 | 72 |  | c.566A>G | p.Tyr189Cys | 4 | 4 |
| 78 |  |  | c.580T>A | p.Cys194Ser | 4 | 4 |
| 79 | 73 |  | c.580T>C | p.Cys194Arg | 4 | 4 |
| 80 | 76 | * | c.581G>A | p.Cys194Tyr | 4 | 4 |
| 81 | 75 |  | c.581G>T | p.Cys194Phe | 4 | 4 |
| 82 | 74 |  | c.581G>C | p.Cys194Ser | 4 | 4 |
| 83 | 77 |  | c.601T>C | p.Cys201Arg | 4 | 5 |
| 84 | 78 |  | c.602G>A | p.Cys201Tyr | 4 | 5 |
| 85 | 79 |  | c.616T>C | p.Cys206Arg | 4 | 5 |
| 86 | 80 |  | c.617G>A | p.Cys206Tyr | 4 | 5 |
| 87 | 81 |  | c.619C>T | p.Arg207Cys | 4 | 5 |
| 88 | 82 |  | c.634T>A | p.Cys212Ser | 4 | 5 |
|  |  | * | c.634T>C | p.Cys212Arg | 4 | 5 |
| 89 |  |  | c.635G>A | p.Cys212Tyr | 4 | 5 |
| 90 |  |  | c.636C>G | p.Cys212Trp | 4 | 5 |
| 91 | 84 |  | c.659A>G | p.Tyr220Cys | 4 | 5 |
| 92 | 85 |  | c.664T>G | p.Cys222Gly | 4 | 5 |
| 93 | 86 |  | c.665G>A | p.Cys222Tyr | 4 | 5 |
| 94 |  |  | c.665G>C | p.Cys222Ser | 4 | 5 |
| 95 | 87 |  | c.671G>A | p.Cys224Tyr | 4 | 5 |
| 96 | 88 | * | c.697T>A | p.Cys233Ser | 5 | 5 |
| 97 |  |  | c.697T>C | p.Cys233Arg | 5 | 5 |
| 98 | 89 |  | c.698G>A | p.Cys233Tyr | 5 | 5 |
| 99 | 90 |  | c.699T>G | p.Cys233Trp | 5 | 5 |
|  | 92 |  | c.714_758del | p.Asp239_Asp253del | 5 | 6 |
| 100 | 93 |  | c.719G>C | p.Cys240Ser | 5 | 6 |
| 101 | 94 |  | c.733T>C | p.Cys245Arg | 5 | 6 |
| 102 | 95 |  | c.733T>A | p.Cys245Ser | 5 | 6 |
|  |  | * | c.734G>A | p.Cys245Tyr |  |  |
| 103 | 96 |  | c.751T>C | p.Cys251Arg | 5 | 6 |
| 104 | 97 |  | c.751T>A | p.Cys251Ser | 5 | 6 |
| 105 | 98 |  | c.751T>G | p.Cys251Gly | 5 | 6 |
| 106 | 99 |  | c.752G>A | p.Cys251Tyr | 5 | 6 |
| 107 | 100 |  | c.773A>G | p.Tyr258Cys | 5 | 6 |
| 108 |  |  | c.778T>G | p.Cys260Gly | 5 | 6 |
| 109 | 101 |  | c.779G>A | p.Cys260Tyr | 5 | 6 |
|  |  | * | c.779G>T | p.Cys260Phe |  |  |
| 110 | 102 |  | c.812G>T | p.Cys271Phe | 6 | 6 |
| 111 |  |  | c.886G>T | p.Gly296Cys | 6 | 7 |
| 112 | 103 |  | c.895A>T | p.Ser299Cys | 6 | 7 |
|  |  | * | c.969C>G | p.Cys323Trp | 6 | 8 |
|  |  | * | c.986G>A | p.Cys329Tyr | 6 | 8 |
| 113 | 104 | * | c.994C>T | p.Arg332Cys | 6 | 8 |
| 114 | 106 |  | c.1004C>G | p.Ser335Cys | 6 | 8 |
| 115 | 107 |  | c.1010A>G | p.Tyr337Cys | 6 | 8 |
| 116 | 108 |  | c.1012T>C | p.Cys338Arg | 6 | 8 |
| 117 |  |  | c.1078T>C | p.Cys360Arg | 7 | 9 |
| 118 |  |  | c.1096T>C | p.Cys366Arg | 7 | 9 |
| 119 | 109 |  | c.1098T>G | p.Cys366Trp | 7 | 9 |
| 120 |  |  | c.1135T>C | p.Cys379Arg | 7 | 9 |
| 121 | 110 |  | c.1136G>C | p.Cys379Ser | 7 | 9 |
| 122 | 111 | * | c.1144G>T | p.Gly382Cys | 7 | 9 |
| 123 | 112 | * | c.1163G>A | p.Cys388Tyr | 7 | 9 |
| 124 | 113 |  | c.1183T>C | p.Cys395Arg | 7 | 10 |
| 125 |  | * | c.1187C>G | p.Ser396Cys | 7 | 10 |
| 126 |  |  | c.1241C>G | p.Ser414Cys | 8 | 10 |
|  |  | * | c.1255T>C | p.Cys419Arg |  |  |
| 127 | 114 |  | c.1258G>T | p.Gly420Cys | 8 | 10 |
| 128 | 115 |  | c.1261C>T | p.Arg421Cys | 8 | 10 |
| 129 |  |  | c.1279C>T | p.Arg427Cys | 8 | 10 |
| 130 | 116 |  | c.1282T>C | p.Cys428Arg | 8 | 10 |
| 131 | 118 |  | c.1283G>A | p.Cys428Tyr | 8 | 10 |
| 132 | 117 |  | c.1283G>C | p.Cys428Ser | 8 | 10 |
|  | 119 |  | c.1300_1308dup | p.Glu434_Leu436dup | 8 | 11 |
| 133 | 120 |  | c.1303T>C | p.Cys435Arg | 8 | 11 |
|  |  | * | c.1304G>A | p.Cys435Tyr | 8 | 11 |
| 134 | 123 |  | c.1318T>A | p.Cys440Ser | 8 | 11 |
| 135 | 121 |  | c.1318T>G | p.Cys440Gly | 8 | 11 |
| 136 | 122 |  | c.1318T>C | p.Cys440Arg | 8 | 11 |
| 137 | 125 |  | c.1337G>C | p.Cys446Ser | 8 | 11 |
| 138 | 124 |  | c.1337G>T | p.Cys446Phe | 8 | 11 |
| 139 | 126 |  | c.1345C>T | p.Arg449Cys | 8 | 11 |
| 140 | 127 | * | c.1363T>C | p.Cys455Arg | 8 | 11 |
| 141 |  |  | c.1364G<A | p.Cys455Tyr | 8 | 11 |
| 142 |  | * | c.1370G>C | p.Cys457Ser | 8 | 11 |
| 143 | 128 |  | c.1394A>G | p.Tyr465Cys | 9 | 11 |
| 144 | 129 |  | c.1450T>G | p.Cys484Gly | 9 | 12 |
| 145 | 130 |  | c.1451G>A | p.Cys484Tyr | 9 | 12 |
| 146 | 131 |  | c.1451G>T | p.Cys484Phe | 9 | 12 |
| 147 | 132 |  | c.1484G>A | p.Cys495Tyr | 9 | 12 |
| 148 |  |  | c.1510T>C | p.Cys504Arg | 10 | 12 |
| 149 | 133 |  | c.1531T>C | p.Cys511Arg | 10 | 13 |
| 150 |  |  | c.1532G>T | p.Cys511Phe | 10 | 13 |
| 151 | 134 |  | c.1532G>A | p.Cys511Tyr | 10 | 13 |
| 152 | 135 |  | c.1582G>T | p.Gly528Cys | 10 | 13 |
| 153 | 136 |  | c.1592G>C | p.Cys531Ser | 10 | 13 |
| 154 | 137 |  | c.1594C>T | p.Arg532Cys | 10 | 13 |
| 155 |  | * | c.1624T>C | p.Cys542Arg | 11 | 13 |
| 156 | 138 | * | c.1625G>A | p.Cys542Tyr | 11 | 13 |
| 157 | 139 | * | c.1630C>T | p.Arg544Cys | 11 | 13-14 |
| 158 | 140 |  | c.1645T>C | p.Cys549Arg | 11 | 14 |
| 159 | 141 |  | c.1646G>A | p.Cys549Tyr | 11 | 14 |
| 160 | 142 |  | c.1672C>T | p.Arg558Cys | 11 | 14 |
| 161 | 143 |  | c.1703G>A | p.Cys568Tyr | 11 | 14 |
| 162 | 144 |  | c.1721A>G | p.Tyr574Cys | 11 | 14 |
| 163 | 146 |  | c.1732C>T | p.Arg578Cys | 11 | 14 |
| 164 |  |  | c.1735T>C | p.Cys579Arg | 11 | 14 |
| 165 | 147 |  | c.1759C>T | p.Arg587Cys | 11 | 15 |
| 166 | 148 |  | c.1771T>C | p.Cys591Arg | 11 | 15 |
| 167 |  |  | c.1774C>T | p.Arg592Cys | 11 | 15 |
| 168 |  |  | c.1790G>C | p.Cys597Ser | 11 | 15 |
| 169 |  |  | c.1816T>C | p.Cys606Arg | 11 | 15 |
|  |  | * | c.1817G>C | p.Cys606Ser | 11 | 15 |
|  |  | * | c.1819C>T | p.Arg607Cys | 11 | 15 |
| 170 | 149 |  | c.1819C>T | p.Arg607Cys | 11 | 15 |
| 171 | 150 |  | c.1918C>T | p.Arg640Cys | 12 | 16 |
| 172 | 151 |  | c.1999G>T | p.Gly667Cys | 13 | 17 |
| 173 |  |  | c.2038C>T | p.Arg680Cys | 13 | 17 |
| 174 |  |  | c.2129A>G | p.Tyr710Cys | 13 | 18 |
| 175 |  |  | c.2149C>T | p.Arg717Cys | 14 | 18 |
| 176 | 152 |  | c.2182C>T | p.Arg728Cys | 14 | 18 |
|  |  | * | c.2185T>G | p.Cys729Gly |  |  |
| 177 | 153 |  | c.2324G>C | p.Cys775Ser | 15 | 20 |
|  | 154 |  | c.2411-1G>T | p.Pro805_Asn856de | inron 15 | 21,22,23 |
| 178 |  |  | c.2815T>C | p.Cys939Arg | 18 | 24 |
| 179 | 155 |  | c.2857G>T | p.Gly953Cys | 18 | 24 |
| 180 |  |  | c.2923G>T | p.Gly975Cys | 18 | 25 |
| 181 | 156 |  | c.2929T>A | p.Cys977Ser | 18 | 25 |
| 182 | 158 |  | c.2951T>G | p.Phe984Cys | 18 | 25 |
| 183 | 159 | * | c.2953C>T | p.Arg985Cys | 18 | 25 |
| 184 | 160 |  | c.2963G>A | p.Cys988Tyr | 18 | 25 |
|  |  | * | c.2963G>T | p.Cys988Phe |  |  |
| 185 | 161 |  | c.2989T>G | p.Cys997Gly | 18 | 25 |
|  |  | * | c.3010T>G | p.Cys1004Gly |  |  |
| 186 | 162 |  | c.3011G>A | p.Cys1004Tyr | 19 | 26 |
| 187 | 163 |  | c.3016C>T | p.Arg1006Cys | 19 | 26 |
| 188 |  |  | c.3037G>T | p.Gly1013Cys | 19 | 26 |
| 189 | 164 |  | c.3043T>C | p.Cys1015Arg | 19 | 26 |
|  |  | * | c.3045C>G | p.Cys1015Trp | 19 | 26 |
| 190 | 165 | * | c.3062A>G | p.Tyr1021Cys | 19 | 26 |
|  |  | * | c.3064T>G | p.Cys1022Gly |  |  |
| 191 |  |  | c.3065G>T | p.Cys1022Phe | 19 | 26 |
| 192 | 167 |  | c.3084G>T | p.Trp1028Cys | 19 | 26 |
| 193 | 168 |  | c.3091C>T | p.Arg1031Cys | 19 | 26 |
| 194 | 169 |  | c.3172G>T | p.Gly1058Cys | 20 | 27 |
| 195 |  |  | c.3182G>A | p.Cys1061Tyr | 20 | 27 |
|  |  | * | c.3200C>G | p.Ser1067Cys | 20 | 27 |
| 196 | 170 |  | c.3206A>G | p.Tyr1069Cys | 20 | 27 |
| 197 | 171 |  | c.3226C>T | p.Arg1076Cys | 20 | 27 |
| 198 | 172 |  | c.3296G>A | p.Cys1099Tyr | 20 | 28 |
| 199 | 173 |  | c.3393C>G | p.Cys1131Trp | 21 | 29 |
| 200 |  | * | c.3427C>T | p.Arg1143Cys | 21 | 29 |
| 201 | 174 |  | c.3691C>T | p.Arg1231Cys | 22 | 31 |
| 202 |  |  | c.3750C>G | p.Cys1250Trp | 23 | 32 |
| 203 | 175 |  | c.3781T>C | p.Cys1261Arg | 23 | 32 |
| 204 | 176 |  | c.3782G>A | p.Cys1261Tyr | 23 | 32 |
| 205 |  |  | c.3893G>T | p.Cys1298Phe | 24 | 33 |
| 206 |  |  | c.3944G>A | p.Cys1315Tyr | 24 | 33 |
