# Supplementary material for: Clinical and Genetic Aspects of CADASIL
Source: Front Aging Neurosci. 2020 May 7;12:91. doi: 10.3389/fnagi.2020.00091 (PMC7224236; doi:10.3389/fnagi.2020.00091)
Supplement: Supplementary file 2 [file Table_2.docx]

# Supplemental Table 2. Original criteria by Davous (a) and new diagnostic criteria for CADASIL in Japan (b).

1. Davous’s criteria (Davous, P. (1998).)

| **Probable** |
| --- |
| **#1 Young age at onset ≤50 years old.** |
| **#2 At least two of the following clinical findings:** |
| **a. Stroke-like episode with a focal neurological deficit.** |
| **b. Migraine** |
| **c. Mood disorder.** |
| **d. Subcortical dementia.** |
| **#3 No vascular risk factor etiologically related to the deficit.** |
| **#4 Evidence of ab inherited autosomal dominant transmission.** |
| **#5 Abnormal MRI imaging of the white matter without cortical infarcts.** |
| Definite |
| **Criteria of probable CADASIL associated with genetic linkage to chromosome 19 (*NOTCH3* mutations) and/or with pathological findings demonstrating small vessel arteriopathy with granular osmiophilic material (GOM).** |
| Possible |
| **#1 Late age at onset >50 years old.** |
| **#2 a. Stroke-like episode without permanent signs.** |
| **b. Minor mood disturbance.** |
| **c. global dementia.** |
| **#3 Minor vascular risk factors such as mild hypertension, mild hyperlipidemia, smoking, use of contraceptive.** |
| **#4 Unknown of incomplete family pedigree.** |
| **#5 Atypical MRI imaging of the white matter.** |
| Exclusion criteria |
| **#1 Age at onset >70 years old.** |
| **#2 Severe hypertension or complicated heart or systemic vascular disease.** |
| **#3 Absence of any other case in a documneted pedigree.** |
| **#5 Normal MRI imaging, age >35** |

1. New diagnostic criteria for CADASIL in Japan (Mizuta I. et al. (2017))

| **Clinical criteria** |
| --- |
| **#1 Age at onset (clinical symptoms #2 or white matter lesions) ≤55 years old.** |
| **#2 At least two of the following clinical findings:** |
| **a. Either of subcortical dementia, long tract signs, or pseudobulbar palsy.** |
| **b. Stroke-like episode with a focal neurological deficit.** |
| **c. Mood disorder.** |
| **d. Migraine.** |
| **#3 Autosomal dominant inheritance.** |
| **#4 White matter lesions involving the anterior temporal pole by MRI or CT.** |
| **#5 Exclusion of leukodystrophy (Adrenoleukodystrophy, metachromatic leukodystrophy, etc.).** |
| Genetic criteria |
| ***NOTCH3* mutations localize in exons 2–24 and result in the gain or loss of cysteine residues in the epidermal growth factor-like repeat domain. Cysteine-sparing variants should be carefully evaluated by skin biopsy and segregation studies** |
| Pathological criteria |
| **The pathological hallmark of CADASIL is granular osmiophilic material (GOM) detected by electron microscopy. Immunostaining of NOTCH3 extracellular domain is also useful.** |
| Definite |
| **CADASIL is definite when the individual fulfills**  (1) **White matter lesions by MRI or CT.**  (2) **Clinical criteria #5**  (3) **Genetic criteria and/or pathological criteria** |
| Probable |
| **CADASIL is probable when the individual fulfills clinical criteria #1–#5.** |
| Possible |
| **CADASIL is possible when the individual has abnormal white matter lesions (Fazekas grade ≥2) and fulfills either of**  (1) **≤55 years old**  (2) **At least one of the symptoms in clinical criteria #2** |

Supplemental Table 3 CADASIL scale-J. (Koizumi T. (2016))

*CADASIL scale-J*

・Without hypertension 5

・Subcortical infarcts 5

・Family history* 5

・Pseudobulbar palsy 3

・Leukoencephalopathy at temporal pole 2

・Age at first onset* ≤ 50 y 2

・Stroke / TIA 　　　　　　　　　　 2

・Without diabetes 1
